# Probiotics and Probiotic-Derived Functional Factors—Mechanistic Insights Into Applications for Intestinal Homeostasis

**DOI:** 10.3389/fimmu.2020.01428

**Published:** 2020-07-03

**Authors:** Fang Yan, D. Brent Polk

**Affiliations:** ^1^Division of Gastroenterology, Hepatology & Nutrition, Department of Pediatrics, Vanderbilt University Medical Center, Nashville, TN, United States; ^2^Department of Pediatrics, Keck School of Medicine of University of Southern California, Los Angeles, CA, United States; ^3^Department of Biochemistry and Molecular Medicine, Keck School of Medicine of University of Southern California, Los Angeles, CA, United States; ^4^Division of Gastroenterology, Hepatology & Nutrition, Children's Hospital Los Angeles, Los Angeles, CA, United States

**Keywords:** gut microbiota, immune response, inflammatory bowel disease, intestinal epithelium, intestinal homeostasis, probiotics, probiotic-derived factor

## Abstract

Advances in our understanding of the contribution of the gut microbiota to human health and the correlation of dysbiosis with diseases, including chronic intestinal conditions such as inflammatory bowel disease (IBD), have driven mechanistic investigations of probiotics in intestinal homeostasis and potential clinical applications. Probiotics have been shown to promote intestinal health by maintaining and restoring epithelial function, ensuring mucosal immune homeostasis, and inhibiting pathogenic bacteria. Recent findings reveal an approach for defining previously unrecognized probiotic-derived soluble factors as potential mechanisms of probiotic action. This review focuses on the impact of probiotics and probiotic-derived functional factors, including probiotic products and metabolites by probiotics, on the cellular responses and signaling pathways involved in maintaining intestinal homeostasis. Although there is limited information regarding the translation of probiotic treatment outcomes from *in vitro* and animal studies to clinical applications, potential approaches for increasing the clinical efficacy of probiotics for IBD, such as those based on probiotic-derived factors, are highlighted in this review. In this era of precision medicine and targeted therapies, more basic, preclinical, and clinical evidence is needed to clarify the efficacy of probiotics in maintaining intestinal health and preventing and treating disease.

## Introduction

The human gastrointestinal tract harbors a broad range of microbiota, which exhibit wide interpersonal differences in taxonomic composition while sharing a functional core set of specific microbial genes and metabolic modules (1, 2). The symbiotic relationship between the gut microbiota and the host establishes an ecosystem that provides a nutrient-rich and metabolically favorable environment for the microbiota, while conferring important benefits to the host for nutrient acquisition and energy balance. Research in humans and animal models has shown that metabolites and functional factors derived from the gut microbiota strongly impact the structural and functional maturation of the gastrointestinal tract, induction of immunotolerance, neurodevelopment and homeostasis of intestinal epithelial cells, and functions of the immune and nervous systems in adulthood [reviewed in (3, 4)].

**Figure 1:**
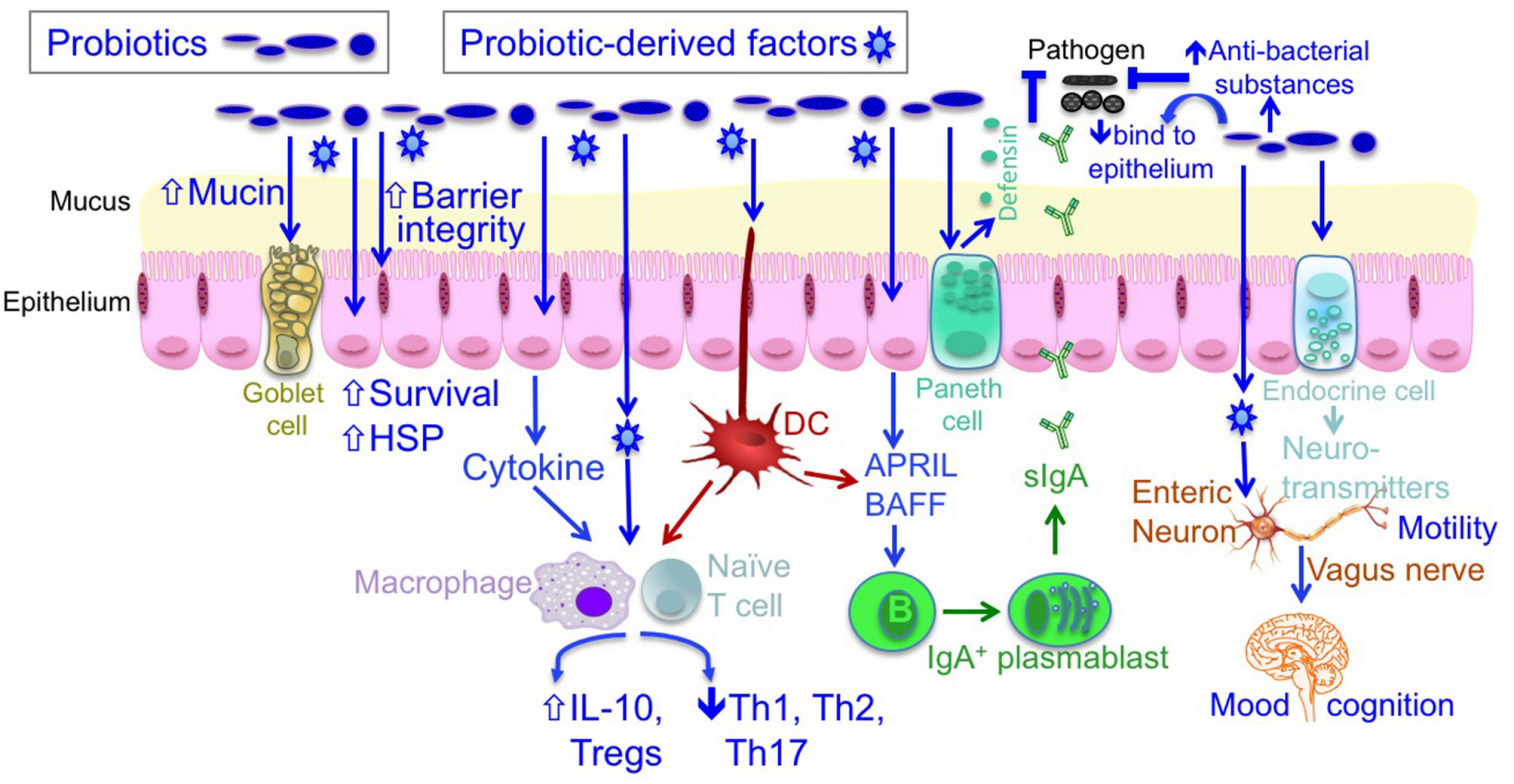
The mechanisms of probiotic action. Probiotics contribute to maintaining homeostasis and prevention and/or treatment of diseases in host, including (1) blocking pathogenic bacterial effects by producing antibacterial substances and competing with pathogens for binding to epithelial cells; (2) promoting intestinal epithelial cell homeostasis by increasing barrier function, mucus production, survival, and cytoprotective responses; (3) defining the balance between necessary and excessive defense immunity by increasing innate immunity, such as production of IgA and defensin, up-regulating anti-inflammatory cytokine production, and inhibiting proinflammatory cytokine production; and (4) regulating the gut-brain axis through production of neurotransmitters and through the vagus nerve. DC, dendritic cell; IL, interleukin; HSP, heat shock protein.

As beneficial microorganisms for host health, probiotics have attracted substantial research interest. The term “probiotics” was originally defined by Lilly and Stillwell as “living microorganisms with low or no pathogenicity that exert beneficial effects on the health of the host” (5). Currently, probiotics are defined as “live microorganisms that, when administered in adequate amounts, confer a health benefit on the host” (6). The most commonly used probiotics include *Bifidobacterium* and *Lactobacillus*. Studies on humans and animal models have revealed distinct cellular and molecular mechanisms of probiotic actions, including the blockage of pathogenic activities via the production of antibacterial substances and competitive inhibition of pathogen and toxin adherence to the intestinal epithelium; the regulation of immune responses via inhibited proinflammatory responses and enhanced anti-inflammatory immunity; the maintenance of intestinal epithelial homeostasis, such as the preservation of barrier structure and function and the blockade of apoptosis in intestinal epithelial cells; and regulation of the gut–brain axis through the production of neurotransmitters and vagus nerve function (6–8) (Figure 1).

**Figure 2:**
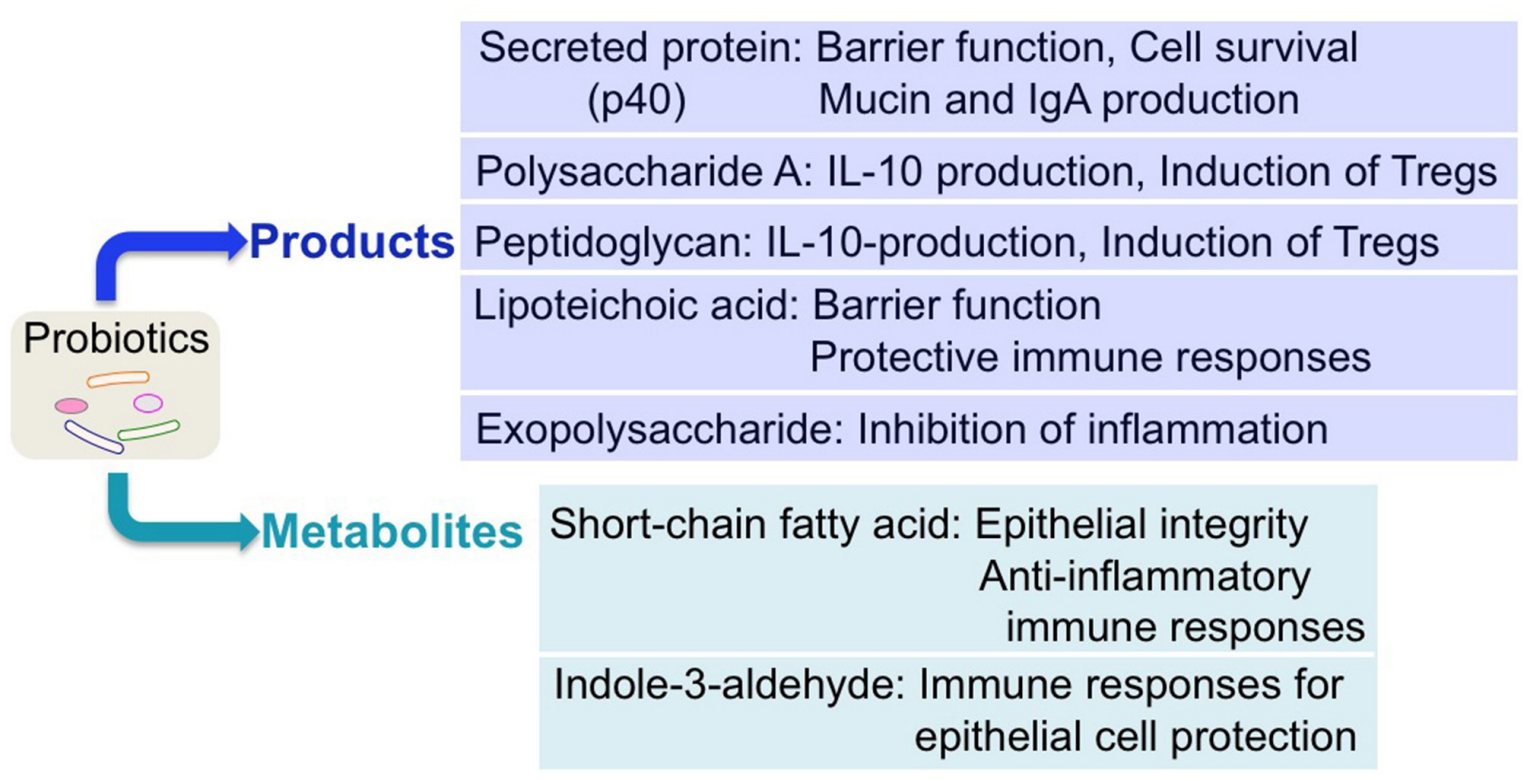
Regulation of host responses by probiotic-derived factors. Several probiotic-derived factors, including products and metabolites, have been identified to exert health-promoting effects on the host. These functional factors of probiotics contribute to reinforcing intestinal barrier function and stimulating anti-inflammatory immune responses, leading to ameliorating intestinal inflammatory disorders.

It is well-known that commensal microorganisms produce variable factors to foster an optimal adaptation to new niches in the host and to directly drive their physiological responses. Importantly, numerous small molecules derived from the human microbiota have been reported to exert biological activities in the host (9). Through efforts to clarify the molecular mechanisms underlying the effects of probiotics, recent research has identified probiotic-derived factors as functional components of probiotics. Notably, a new avenue for elucidating probiotic–host interactions has been discovered, based on the identification of probiotic-derived functional factors, including probiotic products (proteinaceous molecules, carbohydrates, and cell wall components) and metabolites by probiotics (Figure 2). These factors have been shown to regulate host responses and are considered as therapeutic targets.

This review highlights significant research findings on the effect of probiotics on intestinal homeostasis, including the impact of probiotic-derived functional factors on the regulation of intestinal epithelial function, immune responses, and the gut–brain axis. The health-promoting influence of probiotics and probiotic-derived functional factors in intestinal diseases, including inflammatory bowel disease (IBD), colonic cancer and necrotizing enterocolitis, is discussed to support future studies on therapeutic applications of probiotics.

## Intestinal Epithelial Homeostasis

In addition to absorbing nutrients and transporting water and waste products, the intestinal epithelium serves as the first line for the host to discriminate between pathogens and commensal microorganisms in the intestinal tract. Degraded integrity of this monolayer is a major defect in IBD, and mucosal healing is a primary predictor of positive outcomes in this disease (10). Therefore, the regulatory effects of probiotics on intestinal epithelial cells have been widely studied as a mechanism underlying the protection of the intestinal epithelium against inflammation. As summarized in previous reviews (6–8), probiotics regulate the intestinal epithelial functions by maintaining the epithelial barrier, promoting cell survival, stimulating the production of antibacterial substances and cell-protective proteins, enhancing protective immune responses, and inhibiting proinflammatory cytokine production. Many of these responses result from the regulation of specific intracellular signaling pathways by probiotics, such as mitogen-activated protein kinases (MAPK) and nuclear factor (NF)-κB in intestinal epithelial cells (6–8).

The interaction between intestinal epithelial cells and probiotics through probiotic-derived factors has been identified as a previously unrecognized mechanism of action. The finding that *Lactobacillus rhamnosus* GG (LGG) and products recovered from LGG culture broth filtrate can prevent cytokine-induced apoptosis in intestinal and colonic epithelial cell models (11) led to the identification of an LGG-derived soluble protein, p40 (12). p40 has been shown to transactivate the epidermal growth factor (EGF) receptor in intestinal epithelial cells (13) by stimulating the activity of a disintegrin and metalloproteinase 17 (ADAM17) for release of heparin-binding EGF (HB-EGF) (14). Activation of the EGF receptor by p40 is required for p40-induced cytoprotective responses in intestinal epithelial cells, including inhibited cytokine-induced apoptosis, preserved barrier function, and upregulated mucin production in intestinal epithelial cells (13, 15). Furthermore, p40 has been found to upregulate EGF-receptor-dependent production of a proliferation-inducing ligand (APRIL) in intestinal epithelial cells, which may contribute to increased IgA class switching in B cells and enhanced IgA production in the intestine (16). By using a pectin/zein hydrogel bead system to specifically deliver p40 to the colon, p40 can prevent and treat experimental colitis in an EGF-receptor-dependent manner (13).

Short-chain fatty acids generated by metabolizing indigestible carbohydrates from fiber-rich diets by commensal microbiota have long been implicated in a variety of beneficial effects on the host. The production of acetate by *B. longum* subsp. *infantis* 157F contributes to the defense functions of host intestinal epithelial cells for inhibiting translocation of the *Escherichia coli* O157:H7 Shiga toxin from the gut lumen to the blood, thereby protecting mice against death induced by *E. coli* O157:H7 (17). More importantly, an ATP-binding-cassette-type carbohydrate transporter has been identified that confers a probiotic effect on bifidobacterial strains, resulting in increased acetate production (17).

Toll-like receptor (TLR) signaling has been reported as a target of probiotic-derived factors in several studies. LGG and LGG-conditioned media reduce radiation-induced epithelial injury and improve crypt survival through a TLR-2/MyD88 signaling mechanism, leading to repositioning of constitutive COX-2-expressing mesenchymal stem cells to the crypt base (18). It remains unknown whether probiotic-derived factors serve as direct ligands for TLR activation or whether they act through indirect mechanisms. Further studies have revealed that the protective effect of LGG against radiation-induced intestinal epithelial injury is mediated by the production of lipoteichoic acid (LTA), a cell wall polymer in Gram-positive bacteria. LGG-derived LTA fosters the epithelial stem cell niche to protect epithelial stem cells by triggering several adaptive immune responses, including the expression of CXCL12 in macrophages and COX-2-dependent PGE2 secretion from mesenchymal stem cells (19).

In addition to soluble factors, probiotic-derived outer membrane vesicles, such as *E. coli Nissle* 1917 and commensal ECOR63-derived outer membrane vesicles, have been shown to promote barrier function in intestinal epithelial cells (20), and pretreatment of mice with *E. coli Nissle* 1917-derived vesicles has been shown to ameliorate DSS-induced colitis (21). Overall, these studies support the feasibility of applying probiotic-derived products and metabolites as a strategy to promote intestinal health.

In addition to the direct regulation of intestinal epithelial cells by probiotics or probiotic functional factors, probiotics have been found to enhance intestinal epithelial integrity through restoring the balance of the gut microbiota profile. Metabolic disorders are associated with dysbiosis, intestinal inflammation, and disruption of the intestinal barrier function, resulting in leaking of bacterial toxins into the intestinal tract to induce chronic and systemic inflammation. This imbalanced state is referred to as metabolic endotoxemia. The decrease in the abundance of *Akkermansia muciniphila*, a mucin-degrading bacterium in the mucus layer, was observed in adults with obesity (22). Administration of this bacterium to mice with high-fat diet-induced metabolic disorders was able to maintain the gut barrier and inhibit metabolic endotoxemia (23). Interestingly, supplementation with *Bifidobacterium animalis subsp. lactis* 420 to overweight adults caused an increase in *Lactobacillus* and *Akkermansia* and fostered the metabolism toward lean status in a randomized controlled trial (24). These results indicate the importance of the regulatory effects of probiotics on the gut microbiota for maintaining intestinal epithelial homeostasis.

## Protective Mucosal Immune Responses

The identification of probiotic-induced protective immune responses in the host has encouraged the therapeutic application of probiotics in preclinical and clinical studies. To support the application of probiotics, recent studies have explored the mechanisms by which probiotics regulate immune responses. *L. reuteri* stimulates the generation of indole derivatives to activate aryl-hydrocarbon receptor (AhR), leading to the downregulation of Thpok in CD4^+^ intraepithelial lymphocytes (IELs) and reprograming CD4^+^ IELs into CD4^+^CD8αα^+^ IELs (25). A study of SIV-infected macaques demonstrated that VSL#3, Culturelle, and inulin supplementation of antiretroviral treatment increases the reconstitution of colonic CD4^+^ T cells, possibly by increasing APC-related genes in colonic CD45^+^ leukocytes and reducing inflammation-associated fibrosis. Thus, probiotics and prebiotics provide an exciting adjunctive therapeutic approach for enhancing gastrointestinal immune function during HIV infection (26). As another example, the prevention of *Citrobacter rodentium*-induced colitis by probiotics has been studied in mice. *Lactobacillus helveticus* and *L. rhamnosus* have been shown to prevent and treat *C. rodentium*-induced colitis in mice, which is correlated with the downregulation of tumor necrosis factor (TNF)α and interferon (IFN)γ, enhanced transcription of IL-10 and FOXP3, and increased follicular T-regulatory cells (27).

To support thoughtful clinical applications of probiotics, more biological studies of humans or human models are needed. For example, the regulation of immunological tolerance to the microbiota by *Bifidobacterium infantis* 35624 has been studied in humans. *B. infantis*-stimulated human dendritic cells induce Foxp3- and IL-10-secreting T cells, which requires TLR signaling pathways, including TLR-2, TLR-6, and TLR-9 (28). Another study reported *in vitro* effects of a bifidobacterial mixture containing *B. longum, B. breve*, and *B. infantis*, which improved the antigen uptake and processing by DCs obtained from peripheral blood monocytes of pediatric patients with Crohn's disease (CD), but not from ulcerative colitis (UC) and non-IBD controls (29). This type of evidence supports the rationale for adjunctive treatment in human IBD trials with specific probiotics.

The regulation of host immune responses by probiotics and probiotic-derived factors has been highlighted by several recent studies of probiotic–host immune cell interactions. Polysaccharide A (PSA) from outer membrane vesicles of commensal *Bacteroides fragilis* modulates the host's innate immune system through the TLR2 in dendritic cells, resulting in increased regulatory T cells and IL-10 production. This signaling event restricts the activity of T helper 17 (TH17) cells and promotes *B. fragilis* colonization in germ-free mice (30) and induces immunomodulatory effects and prevents experimental colitis (31). Interestingly, PSA in outer membrane vesicle-induced signaling in DCs requires IBD-associated genes, ATG16L1 and NOD2, to activate a non-canonical autophagy pathway during protection from colitis (32). This evidence suggests that the functions of commensal bacterium-derived factors are affected by host genetics, highlighting important relationships between polymorphisms in susceptibility genes and protective microbial effects on the host, which may extend to probiotic-induced host responses.

Peptidoglycan (PGN) has been identified as an active compound in probiotic functionality. PGN purified from *Lactobacillus salivarius* Ls33, a strain that has protective effects in colitis, induces IL-10-producing DCs in a NOD2-dependent manner *in vitro* and protects mice from colitis in an IL-10-dependent manner. PGN also promotes the development of CD103^+^ DCs and CD4^+^Foxp3^+^ regulatory T cells. However, a non-anti-inflammatory strain of *L. salivarius-*derived PGN did not show these effects. Structural analysis suggests that PGNs from anti-inflammatory strains contain a muropeptide, M-tri-Lys, that mediates the protective roles of PGNs in colitis in a NOD2-dependent, but MyD88-independent manner (33).

Probiotic surface layer proteins, such as exopolysaccharide (EPS) molecules, also play pivotal and beneficial roles in probiotic–host interactions. EPS on the cell surface of *B. breve* UCC2003 provides stress tolerance and promotes *in vivo* persistence, but not initial colonization. EPS is also involved in modulating the ability of commensal bacteria to remain immunologically silent. However, EPS can reduce the colonization levels of gut pathogens, such as *Citrobacter rodentium* (34). Another surface layer protein, surface layer protein A (SlpA), on *Lactobacillus acidophilus* NCK2187 has been shown to bind to the C-type lectin SIGNR3 to exert regulatory signals, leading to the mitigation of colitis, maintenance of healthy gastrointestinal microbiota, and protected gut mucosal barrier function (35). These results provide critical insights into the potential development of probiotic or commensal-derived factors in the treatment of intestinal inflammatory disorders.

In contrast to probiotic-derived protective factors, LTA is considered as the Gram-positive equivalent of Gram-negative LPS in stimulating immune responses. Deletion of the phosphoglycerol transferase gene that is responsible for LTA biosynthesis in *L. acidophilus* NCK2025 significantly reduces colonic polyps and systemic inflammation and increases anti-inflammatory regulatory T cells in mice with conditional APC gene truncation in colonic and ileal epithelial cells. Thus, this study reveals the proinflammatory role of LTA (36).

Probiotic metabolites have also been reported to exert protective effects on the intestinal epithelium. A *Lactobacillus reuteri* D8 metabolite, indole-3-aldehyde, can stimulate lamina propria lymphocytes to secret IL-22 through AhR, leading to phosphorylation of STAT3 to accelerate intestinal epithelial cell proliferation, thus recovering damaged intestinal mucosa (37).

These results support the involvement of probiotic-derived factors in active communication between probiotics and host immune cells for signal regulation in immune cells relevant to innate immunity under healthy conditions and in adaptive immune responses to disease.

## The Gut–Brain Axis

The first evidence to support the involvement of the gut microbiota in the gut–brain axis came from studies showing abnormal neurotransmitter and brain-derived neurotrophic factor levels, reduced anxiety responses, and increased motor activity in germ-free mice compared to those with intact bacterial communities (37). Subsequent research revealed that microbiota-derived short-chain fatty acids and fermentation products promote microglia maturation and function (38), and a commensal bacterium, *B. fragilis*, restores the neurological function (39). Furthermore, a mutation in KDM5, a histone demethylase, has been found in intellectual disability (ID) and autism spectrum disorder (ASD) patients. A previous study demonstrated that KDM5 deficiency in *Drosophila melanogaster* induces intestinal barrier dysfunction and changes in social behavior that correlate with alteration of the gut microbiota, which can be partially rescued by a probiotic *Lactobacillus* strain (40). These findings indicate the need to further study probiotics that may influence the gut–brain axis.

One pathway of gut–brain communication occurs through activation of the vagus nerve as part of the parasympathetic nervous system. The involvement of probiotics in regulation of the vagus nerve function is supported by several studies. In a mouse model of chemically induced colitis, *B. longum* colonization in the gut reduced anxiety-like behavior via activation of the vagal pathway, independently of brain-derived neurotrophic factor production (41). In mice, *L. rhamnosus* supplementation has been shown to modulate the expression of γ-aminobutyric acid receptors in the brain, thus affecting signaling of the major inhibitory neurotransmitter. Consequently, this microbe-dependent communication through the vagus nerve can ameliorate stress and anxiety- and depression-related behavior (42). Moreover, probiotic supplementation has been shown to markedly change behavior in rodents, with correlated changes in central neurochemistry. *L. helveticus* NS8 improves anxiety, depression, and cognitive dysfunction in rats with chronic restraint stress. *L. helveticus* NS8 also reduces plasma corticosterone and adrenocorticotropic hormone levels, increases plasma IL-10 levels, and restores hippocampal serotonin and norepinephrine levels and hippocampal brain-derived neurotrophic factor mRNA expression in chronic stress rats. These results indicate an antidepressant effect of *L. helveticus* NS8 in rats subjected to chronic restraint stress depression, an effect that may be due to the microbiota–gut–brain axis (43).

Dysbiosis has also been linked to changes in cognitive activity and behavior, such as the pathophysiology of stress-related disorders, anxiety, and autism in humans (44). Thus, the effects of probiotics on neurological function and associated disorders have been examined in humans. A randomized, double-blinded, placebo-controlled trial showed that 4-week *B. longum* 1714™ administration regulated resting neural activities related to enhanced vitality and reduced mental fatigue as well as neural responses during social stress, including the activation of brain coping centers to counter-regulate negative emotions (40). Consumption of fermented milk products containing seven probiotic strains of *Bifidobacterium, Lactobacillus*, and *Lactococcus* for 4 weeks affected the activity of brain regions that control the central processing of emotion and sensation in healthy women (45). Furthermore, a systematic review of 10 randomized controlled trials showed that probiotic supplementation can positively affect anxiety and depressive symptoms in humans (46). Thus, the impact of probiotics on the gut–brain axis is a potential area for studies on broad probiotic applications.

## Intestinal Development and Diseases in Early Life

While colonization of the intestinal microbiota in humans may begin *in utero* (47), it is significantly influenced by microbial exposures at birth (48). Strong evidence supports the contribution of microbiota colonization of the gastrointestinal tract to functional maturation of the intestinal tract (49) and the development of immunity in early life (50). Therefore, the impact of the probiotics on early life has attracted significant research interest.

Effects of probiotics on intestinal development have been observed. Neonatal LGG colonization enhances intestinal functional maturation, including intestinal epithelial cell proliferation, differentiation, and tight junction formation, and increases the diversity and richness of the colonic mucosa-associated microbiota prior to weaning in mice (51). Both live and heat-killed LGG have been reported to enhance maturation of the intestinal barrier function in 7-day-old conventionally raised mice receiving treatment from postnatal day 1–7 (52). Furthermore, *L. reuteri* DSM 17938 has been found to stimulate enterocyte proliferation and migration in 8-day-old neonatal mice treated from day 5–8 (53). In addition, monocolonization of *Lactobacillus plantarum* enhanced growth in an infant germ-free mouse model of chronic undernutrition and growth failure (54).

Supplementation with probiotics in formula milk for disease prevention and treatment is becoming increasingly common. Clinical studies have revealed strong evidence for treatment of infants and young children with infectious diarrhea (55, 56) and for prevention of antibiotic-associated diarrhea in children (56); thus, probiotics are highly recommended for use in these cases (57, 58).

However, the efficacy of probiotics for the prevention of necrotizing enterocolitis (NEC) in premature infants are not consistent. A double-blind randomized placebo-controlled trial including 1,310 babies with the gestational ages of 23–30 weeks found no difference of late-onset sepsis, NEC stage 2 or above or death of babies receiving *Bifidobacterium breve* strain BBG-001, as compared to babies with placebo (59). Another randomized placebo-controlled trial including 1,099 babies born early than 32 weeks completed gestation reported that supplementation of a probiotic combination containing *B. infantis, Streptococcus thermophiles*, and *Bifidobacterium lactis* did not affect the rates of late onset sepsis or mortality, but significantly reduced the incidence of NEC (60).

Evidence of a potential impact of administering probiotics to pregnant women on reducing the risk of newborns with NEC has been reported. A meta-analysis of randomized controlled trials including 18 randomized controlled trials with 4,356 pregnant women showed that the risks of death and NEC were significantly reduced in pregnant women receiving probiotics (61). This evidence was also reported by another systematic review that concluded the risk of NEC was decreased in women at risk of preterm birth receiving probiotics and antenatal corticosteroids (62).

Although the clinical efficacy of probiotics for prevention of NEC is uncertain, results from animal models and *in vitro* experiments provide some interesting insights into the effects of probiotics on NEC. *L. rhamnosus* HN001 has been shown to attenuate NEC severity in newborn mice and premature piglets in a TLR-9-dependent manner, and DNA from *L. rhamnosus* HN001 reduces the extent of proinflammatory signaling in cultured enterocytes (63). Furthermore, *B. infantis*-conditioned medium protects against *Cronobacter sakazakii*-induced intestinal inflammation in newborn mice and inhibits interleukin (IL)-1β-induced IL-6 induction in immature enterocytes via regulation of TLR-4 signaling (64). Further, indole-3-lactic acid inhibits IL-1β-stimulated IL-8 production in immature enterocytes (65). This evidence suggests that probiotic-derived factors may aid in protection of immature epithelium against inflammation.

The supplementation with probiotics and probiotic-derived functional factors in early life has potential for promoting growth and preventing some diseases. Thus, long-term health outcomes of supplementation with probiotics and functional factors in early life should be elucidated for expanding the application of probiotics for human health.

## Intestinal Inflammatory Diseases and Cancer

### Inflammatory Bowel Disease (IBD)

IBD, consisting of UC and CD, results from a complex interaction among an altered gut microbial community, environmental factors, and inappropriate mucosal immune responses in genetically susceptible individuals (66). IBD has become a public health challenge, with accelerating incidence worldwide (67). Current treatments for IBD, including 5-aminosalicylic acid [5-ASA] preparations, corticosteroids, and immunosuppressive and biological therapies, such as the use of anti-TNF agents, generally induce sustained remission in less than half of patients (68). Therefore, new therapeutic approaches remain an unmet need in IBD research. Dysbiosis of the gut microbiota has been identified in patients with IBD, although it remains unclear whether dysbiosis is the cause or a consequence of other factors associated with this disease (69, 70). Therefore, manipulation of the gut microbiota, such as that induced by probiotic supplementation, has been under investigation for prevention and treatment of IBD.

Although probiotics have been recommended for adjunctive therapy for inducing and maintaining a remission of pouchitis and UC (57, 58), the clinical efficacy of probiotics for inducing and maintaining a remission of UC is limited. Results from a recent review analyzing 14 randomized controlled trials including 865 participants show low-certainty evidence that probiotics induce clinical remission in active UC patients when compared with a placebo and little or no difference when compared with 5-ASA (71). Based on limited information, this review reported that the combination of probiotics and 5-ASA may slightly improve the induction of remission compared with 5-ASA alone in patients with mild, but not severe, disease (71). In fact, the combination of probiotics with traditional IBD therapy is the most widely investigated treatment for UC patients in current clinical trials. VSL#3, a probiotic preparation including *Lactobacillus, Bifidobacterium*, and *Streptococcus* strains, was administered to children with newly diagnosed UC who received co-treatment of steroid induction and mesalamine maintenance in a prospective placebo-controlled trial. This study showed that VSL#3 improved the maintenance of remission (72). In adult patients with mild to moderate UC who did not respond to conventional therapy, beneficial effects of VSL#3 on remission induction were observed in patients with concomitant therapy of mesalamine and corticosteroids (73). In assessing the effects of probiotics on maintaining remission in UC patients, a double-blind trial showed that *E. coli Nissle* 1917 treatment and mesalazine treatment had equal efficacy (74). In addition, as adjunctive treatment with 5-ASA and/or immunosuppressants, VSL#3 reduced disease activity in patients with relapsing mild to moderate UC in a double-blind, randomized, placebo-controlled study (75). Pouchitis is a complication after ileal pouch-anal anastomosis for UC treatment. Clinical studies have provided evidence supporting the use of VSL#3 to reduce the likelihood of relapse after ileal pouch-anal anastomosis for UC patients (76).

In contrast, studies have revealed that probiotics have little beneficial effect on CD. For CD patients in which the diseased gut had been removed, results from a randomized controlled trial suggested that LGG treatment for 12 months did not prevent endoscopic recurrence or reduce the severity of recurrent lesions (77). In addition, *Lactobacillus johnsonii* LA1 did not result in any improvements for endoscopic recurrence of CD in patients after surgical resection of lesions in a randomized, double-blind, placebo-controlled trial (78) or a multicenter, randomized, controlled clinical trial (79). Current clinical evidence suggest that probiotics, including LGG, *Lactobacillus casei, B. breve, B. longum, E. coli Nissle*, and *Saccharomyces boulardii*, alone or in combination with prebiotic preparations do not strengthen the effects of conventional therapies in inducing or maintaining remission over placebos in patients with CD [reviewed in (80)].

### Colorectal Cancer

Dysbiosis has been implicated as a risk factor or a consequence of carcinogenesis in patients with colorectal cancer (81). Alterations in the composition, distribution, or metabolism of the microbiota in the colon may produce an environment in the colon that promotes inflammation, dysplasia, and cancer (82). Using several animal models of colon cancer, such as azoxymethane (AOM)-dextran sodium sulfate, IL-10 knock-out, and 1,2-dimethylhydrazine-induced colon cancer in mice and rats, studies have suggested that probiotics may inhibit cancer development and progression by inactivating mutagens or carcinogens, modulating intestinal microflora and their metabolism, inducing apoptosis, and inhibiting tumor cell differentiation by suppressing tumor-promoting signaling pathways and immunomodulation [reviewed in (83, 84)]. To elucidate the molecular mechanisms of probiotic action in the prevention of tumor development, one study revealed a *L. casei* ATCC334-produced tumor-suppressive molecule, ferrichrome. This molecule stimulates the ER stress response and c-jun N-terminal kinase (JNK) signaling though upregulation of DDIT3 gene expression, thus inhibiting growth and promoting apoptosis in colonic tumor cell lines and in a xenograft model, as reported in (85). These results emphasize the importance of investigating probiotic-derived factors as potential therapeutic targets.

Research has raised interesting issues regarding the roles of probiotics in different mouse models of colonic cancer. Administration of VSL#3, a probiotic mixture, reduced chronic inflammation and prevented or delayed the development of dysplasia and carcinoma in a model of AOM-DSS-induced chronic colitis-associated cancer in wild-type mice (86). In an AOM-IL10^−/−^ mouse model of colitis-associated colon cancer, treatment with VSL#3 did not protect against inflammation or tumorigenesis. Instead, VSL#3 significantly enhanced tumor penetrance, multiplicity, histologic dysplasia scores, and adenocarcinoma invasion relative to non-VSL#3-treated mice. VLS#3 treatment also altered the luminal and mucosally adherent microbiota in this model (87). Therefore, mechanic studies of individual bacteria on cancer development are needed to elucidate these different responses in tumor development upon VSL#3 administration.

Information from clinical trials on probiotics in cancer prevention and treatment is limited. Several clinical studies have suggested some potential roles of probiotics in suppressing colon cancer development. In a randomized trial, *L. casei* and dietary fiber significantly reduced the tumor occurrence rate after 4 years of treatment for patients in whom at least two colorectal tumors with a grade of moderate atypia or higher had been removed (88). Another randomized, double-blind, placebo-controlled trial showed that LGG and *B. lactis* Bb12 combined with inulin treatment for 12 weeks in colon cancer and polypectomized patients reduced cancer risk factors, including a modulated gut microbiota, reduced colorectal proliferation, improved epithelial barrier function, reduced IL-2 secretion, and increased IFN-γ production (89). In probiotic research on cancer prevention and treatment, results from two melanoma studies have provided exciting evidence. *Bifidobacterium* increases the therapeutic effects of antibodies targeting the PD-1/PD-L1 therapeutic axis (90) while *B. fragilis* augments the immunostimulatory effects of CTLA-4 blockade (91). Thus, combinations of immunotherapy and probiotics for colon cancer treatment may merit further investigation.

## Ppotential Approaches to Increase Probiotic Efficacy

It has been challenging for prior clinical trials to assess the bioavailability and biopharmacology of probiotics in the gastrointestinal tract. Enhancing *in vivo* probiotic viability by encapsulation may improve probiotic efficacy. For example, encapsulation of LGG in hydrogel beads, prepared using pectin, glucose, and calcium chloride and lyophilized by freeze-drying, increases the survival rate and growth of LGG under low-acid conditions and enzyme digestion conditions, thereby enhancing the ability of LGG to prevent colitis (92).

In addition, studies defining dosing and delivery are needed to clarify the efficacy of probiotic application as a unique biological therapy. Current results from human studies suggest that probiotic survival in the host gastrointestinal tract is dose- and strain-dependent. Most probiotics can only be recovered from feces within 1–2 weeks after consumption has ceased; thus, permanent colonization of probiotic strains in the adult gut does not occur or occurs at very low rates (93). Environmental factors in the host have been shown to affect probiotic colonization. *B. longum* AH1206 engraftment is associated with a low abundance of resident *B. longum* and an under-representation of specific carbohydrate utilization genes in the pre-treatment microbiome (94).

Because the microbial exposure at birth shapes the acquisition and structure of the initial microbiota in newborns (95) and because there is a narrow time window for colonization (96), early exposure to probiotics may enhance probiotic colonization. A model of conventionally raised mice with neonatal LGG colonization was generated to explore the nature of neonatal probiotic colonization in the host. In this model, colonization with LGG is age-dependent, with the highest colonization rates occurring in mice receiving LGG from postnatal day 1–5 (51).

Precision medicine is expected to address unmet therapeutic needs by offering the best available treatments to patients in their disease course. A recent human study explored interesting evidence that reconstitution of the indigenous fecal microbiome and recovery of gut transcriptome toward homeostatic status after antibiotic treatment was delayed by multiple strains of probiotic supplementation and enhanced by autologous fecal microbiome transplantation, although colonization of probiotics in the gut was enhanced by the antibiotic treatment. Remarkably, soluble factors from these probiotics inhibited growth of the human fecal microbiota *in vitro*, which may contribute to impairing the post-antibiotic probiotic benefits (97). This evidence suggests that personalized probiotic approaches are needed for probiotics to exert their beneficial effects without affecting other biological events in the host.

Current therapies for IBD do not target the intestinal epithelium, where the primary deficits occur in a subset of patients (98). Thus, the potential role of probiotics in an era of precision medicine is highly attractive for research. To understand the differences between therapeutic potential and actual clinical outcomes of probiotic use in IBD, several issues should be considered. It is uncertain whether probiotics exert the same functions in IBD patients as in healthy controls. Loss of the ability to recognize and/or kill gut microbes is a characteristic associated with the genetic polymorphisms in IBD (99). Thus, exogenous agents, such as probiotics, may not induce responses in IBD patients as they do in *in vitro* experiments or animal models. Instead, IBD patients may respond better to protective bacteria, such as *Faecalibacterium prausnitzii*, a normal flora member under physiological conditions that is reduced in IBD (100–102). Therefore, developing personalized probiotic treatments with selected probiotics based on individual alterations in microbial profile and activities, mucosal injury, and abnormal epithelial and immune responses may open a new line of therapy in the future.

## Future Studies

Combined with bioinformatics, rapidly developing genomic approaches have been applied to identify biosynthetic genes and to predict the structure and functions of the gut microbiota. These advances facilitate research exploring the genomic features of probiotic bacteria. For example, a comparative genomic study identified 73 genes responsible for cell growth and replication, constituting a core genome for the *Lactobacillus* family, by analyzing *Lactobacillus* genomes and other genomes associated with *Lactobacilli*. The definitive resource for mining *Lactobacillus* contains genes modifying carbohydrates, proteins, and other macromolecules and novel clustered regularly interspaced short palindromic repeats (CRISPR)-CRISPR-associated protein (Cas) systems (103). Thus, the use of genome sequence mining, comparative genomics, and metagenomics to study the biological functions of predicted metabolic products of probiotics may represent a new line of needed investigation.

One of the challenges in probiotic research arises because probiotics are not created equally. Notably, interpersonal variabilities in environmental factors, such as diet and microbiome, are often neglected in study design and analysis for probiotic research. It is well-known that host genetics and the environment affect the composition and function of the gut microbiota (104, 105). As components of the gut microbiota, the functions of probiotics are also likely to be associated with host genetics and environmental factors. Currently, interactions between probiotics and host factors are being considered in studies on probiotic-mediated effects. The probiotic mixture VSL#3 treatment shows mouse strain-specific alterations in immunologic phenotype under homeostasis conditions, suggesting that the effects of probiotics depend on the genetic background of the host (106). Thus, host factors should be considered when designing studies and evaluating results. Efforts focused on personalized probiotic applications for intestinal homeostasis maintenance and for disease prevention and treatment will be crucial in improving the efficacy of probiotics. Likewise, findings from such studies may explain the wide variability in responses to probiotics in intestinal disease.

Synthetic biology approaches have also been applied for disease diagnosis and therapy. *E. coli Nissle* 1917 has been used to develop an orally administered diagnostic reagent that can produce detectable signals in urine to identify liver metastasis in mice (107). In addition, two engineered *E. coli Nissle* 1917 strains have been generated; one enhances wound healing in human intestinal epithelial cells by secreting EGF (108), and the other protects the intestinal epithelium from injury in dextran sodium sulfate-induced colitis in mice by creating fibrous matrices consisting of trefoil factors (109). Thus, future applications of probiotics may differ from those indicated by previous clinical trials.

In conclusion, the current evidence highlights the potential of probiotics and probiotic-derived factors in supporting host homeostasis, with potential for human health and disease prevention and treatment. However, precise mechanistic data are needed to select specific probiotics for well-designed and appropriately powered clinical trials. The availability of biomarkers and specific surrogates of anticipated outcomes would likewise accelerate studies for disease prevention and treatment. Furthermore, the use of probiotics as carriers for directly delivering anti-inflammatory and intestinal epithelial repair factors to the intestinal tract may serve as a distinct advance in future probiotic applications.

## Author Contributions

FY and DP contributed to the drafting and editing of this article. Both authors contributed to the article and approved the submitted version.

## Conflict of Interest

The authors declare that the research was conducted in the absence of any commercial or financial relationships that could be construed as a potential conflict of interest.

## References

[1] Human Microbiome Project C. Structure, function and diversity of the healthy human microbiome. Nature. (2012) 486:207–14. 10.1038/nature11234 22699609 PMC3564958

[2] Lloyd-Price J Abu-Ali G Huttenhower C. The healthy human microbiome. Genome Med. (2016) 8:51. 10.1186/s13073-016-0307-y 27122046 PMC4848870

[3] Sharon G Sampson TR Geschwind DH Mazmanian SK. The central nervous system and the gut microbiome. Cell. (2016) 167:915–32. 10.1016/j.cell.2016.10.027 27814521 PMC5127403

[4] Thaiss CA Zmora N Levy M Elinav E. The microbiome and innate immunity. Nature. (2016) 535:65–74. 10.1038/nature18847 27383981

[5] Lilly DM Stillwell RH. Probiotics: growth-promoting factors produced by microorganisms. Science. (1965) 147:747–8. 10.1126/science.147.3659.747 14242024

[6] Hill C Guarner F Reid G Gibson GR Merenstein DJ Pot B. Expert consensus document. The international scientific association for probiotics and prebiotics consensus statement on the scope and appropriate use of the term probiotic. Nat Rev Gastroenterol Hepatol. (2014) 11:506–14. 10.1038/nrgastro.2014.66 24912386

[7] Thomas CM Versalovic J. Probiotics-host communication: modulation of signaling pathways in the intestine. Gut Microbes. (2010) 1:148–63. 10.4161/gmic.1.3.11712 20672012 PMC2909492

[8] Vanderpool C Yan F Polk DB. Mechanisms of probiotic action: implications for therapeutic applications in inflammatory bowel diseases. Inflamm Bowel Dis. (2008) 14:1585–96. 10.1002/ibd.20525 18623173

[9] Donia MS Fischbach MA. Human microbiota. Small molecules from the human microbiota. Science. (2015) 349:1254766. 10.1126/science.1254766 26206939 PMC4641445

[10] Baert F Moortgat L Van Assche G Caenepeel P Vergauwe P De Vos M. Mucosal healing predicts sustained clinical remission in patients with early-stage crohn's disease. Gastroenterology. (2010) 138:463–8. 10.1053/j.gastro.2009.09.056 19818785

[11] Yan F Polk DB. Probiotic bacterium prevents cytokine-induced apoptosis in intestinal epithelial cells. J Biol Chem. (2002) 277:50959–65. 10.1074/jbc.M207050200 12393915 PMC4006994

[12] Yan F Cao H Cover TL Whitehead R Washington MK Polk DB. Soluble proteins produced by probiotic bacteria regulate intestinal epithelial cell survival and growth. Gastroenterology. (2007) 132:562–75. 10.1053/j.gastro.2006.11.022 17258729 PMC3036990

[13] Yan F Cao H Cover TL Washington MK Shi Y Liu L. Colon-specific delivery of a probiotic-derived soluble protein ameliorates intestinal inflammation in mice through an EGFR-dependent mechanism. J Clin Invest. (2011) 121:2242–53. 10.1172/JCI44031 21606592 PMC3104743

[14] Yan F Liu L Dempsey PJ Tsai YH Raines EW Wilson CL. A lactobacillus rhamnosus GG-derived soluble protein, p40, stimulates ligand release from intestinal epithelial cells to transactivate epidermal growth factor receptor. J Biol Chem. (2013) 288:30742–51. 10.1074/jbc.M113.492397 24043629 PMC3798544

[15] Wang L Cao H Liu L Wang B Walker WA Acra SA. Activation of epidermal growth factor receptor mediates mucin production stimulated by p40, a lactobacillus rhamnosus GG-derived protein. J Biol Chem. (2014) 289:20234–44. 10.1074/jbc.M114.553800 24895124 PMC4106339

[16] Wang Y Liu L Moore DJ Shen X Peek RM Acra SA. An LGG-derived protein promotes IgA production through upregulation of APRIL expression in intestinal epithelial cells. Mucosal Immunol. (2016) 10:373–84. 10.1038/mi.2016.57 27353252 PMC5199635

[17] Fukuda S Toh H Hase K Oshima K Nakanishi Y Yoshimura K. Bifidobacteria can protect from enteropathogenic infection through production of acetate. Nature. (2011) 469:543–47. 10.1038/nature09646 21270894

[18] Ciorba MA Riehl TE Rao MS Moon C Ee X Nava GM. Lactobacillus probiotic protects intestinal epithelium from radiation injury in a TLR-2/cyclo-oxygenase-2-dependent manner. Gut. (2011) 61:829–38. 10.1136/gutjnl-2011-300367 22027478 PMC3345937

[19] Riehl TE Alvarado D Ee X Zuckerman A Foster L Kapoor V. *Lactobacillus rhamnosus* GG protects the intestinal epithelium from radiation injury through release of lipoteichoic acid, macrophage activation and the migration of mesenchymal stem cells. Gut. (2019) 68:1003–13. 10.1136/gutjnl-2018-316226 29934438 PMC7202371

[20] Alvarez CS Badia J Bosch M Gimenez R Baldoma L. Outer membrane vesicles and soluble factors released by probiotic *Escherichia coli* nissle 1917 and commensal ECOR63 enhance barrier function by regulating expression of tight junction proteins in intestinal epithelial cells. Front Microbiol. (2016) 7:1981. 10.3389/fmicb.2016.01981 28018313 PMC5156689

[21] Fabrega MJ Rodriguez-Nogales A Garrido-Mesa J Algieri F Badia J Gimenez R. Intestinal anti-inflammatory effects of outer membrane vesicles from *Escherichia coli* nissle 1917 in DSS-experimental colitis in mice. Front Microbiol. (2017) 8:1274. 10.3389/fmicb.2017.01274 28744268 PMC5504144

[22] Crovesy L Masterson D Rosado EL. Profile of the gut microbiota of adults with obesity: a systematic review. Eur J Clin Nutr. (2020). 10.1038/s41430-020-0607-6. [Epub ahead of print]. 32231226

[23] Everard A Belzer C Geurts L Ouwerkerk JP Druart C Bindels LB. Cross-talk between akkermansia muciniphila and intestinal epithelium controls diet-induced obesity. Proc Natl Acad Sci USA. (2013) 110:9066–71. 10.1073/pnas.1219451110 23671105 PMC3670398

[24] Hibberd AA Yde CC Ziegler ML Honore AH Saarinen MT Lahtinen S. Probiotic or synbiotic alters the gut microbiota and metabolism in a randomised controlled trial of weight management in overweight adults. Benef Microbes. (2019) 10:121–35. 10.3920/BM2018.0028 30525950

[25] Cervantes-Barragan L Chai JN Tianero MD DiLuccia B Ahern PP Merriman J. Lactobacillus reuteri induces gut intraepithelial CD4^+^CD8α*α*^+^ T cells. Science. (2017) 357:806–10. 10.1126/science.aah5825 28775213 PMC5687812

[26] Klatt NR Canary LA Sun X Vinton CL Funderburg NT Morcock DR. Probiotic/prebiotic supplementation of antiretrovirals improves gastrointestinal immunity in SIV-infected macaques. J Clin Invest. (2013) 123:903–7. 10.1172/JCI66227 23321668 PMC3561826

[27] Rodrigues DM Sousa AJ Johnson-Henry KC Sherman PM Gareau MG. Probiotics are effective for the prevention and treatment of citrobacter rodentium-induced colitis in mice. J Infect Dis. (2012) 206:99–109. 10.1093/infdis/jis177 22430833

[28] Konieczna P Groeger D Ziegler M Frei R Ferstl R Shanahan F. Bifidobacterium infantis 35624 administration induces Foxp3 T regulatory cells in human peripheral blood: potential role for myeloid and plasmacytoid dendritic cells. Gut. (2012) 61:354–66. 10.1136/gutjnl-2011-300936 22052061

[29] Strisciuglio C Miele E Giugliano FP Vitale S Andreozzi M Vitale A. Bifidobacteria enhance antigen sampling and processing by dendritic cells in pediatric inflammatory bowel disease. Inflamm Bowel Dis. (2015) 21:1491–8. 10.1097/MIB.0000000000000389 25895109

[30] Round JL Lee SM Li J Tran G Jabri B Chatila TA. The Toll-like receptor 2 pathway establishes colonization by a commensal of the human microbiota. Science. (2011) 332:974–77. 10.1126/science.1206095 21512004 PMC3164325

[31] Shen Y Giardino Torchia ML Lawson GW Karp CL Ashwell JD Mazmanian SK. Outer membrane vesicles of a human commensal mediate immune regulation and disease protection. Cell Host Microbe. (2012) 12:509–20. 10.1016/j.chom.2012.08.004 22999859 PMC3895402

[32] Chu H Khosravi A Kusumawardhani IP Kwon AH Vasconcelos AC Cunha LD. Gene-microbiota interactions contribute to the pathogenesis of inflammatory bowel disease. Science. (2016) 352:1116–20. 10.1126/science.aad9948 27230380 PMC4996125

[33] Macho Fernandez E Valenti V Rockel C Hermann C Pot B Boneca IG. Anti-inflammatory capacity of selected lactobacilli in experimental colitis is driven by NOD2-mediated recognition of a specific peptidoglycan-derived muropeptide. Gut. (2011) 60:1050–59. 10.1136/gut.2010.232918 21471573

[34] Fanning S Hall LJ Cronin M Zomer A MacSharry J Goulding D. Bifidobacterial surface-exopolysaccharide facilitates commensal-host interaction through immune modulation and pathogen protection. Proc Natl Acad Sci USA. (2012) 109:2108–13. 10.1073/pnas.1115621109 22308390 PMC3277520

[35] Lightfoot YL Selle K Yang T Goh YJ Sahay B Zadeh M. SIGNR3-dependent immune regulation by Lactobacillus acidophilus surface layer protein A in colitis. EMBO J. (2015) 34:881–95. 10.15252/embj.201490296 25666591 PMC4388597

[36] Khazaie K Zadeh M Khan MW Bere P Gounari F Dennis K. Abating colon cancer polyposis by Lactobacillus acidophilus deficient in lipoteichoic acid. Proc Natl Acad Sci USA. (2012) 109:10462–67. 10.1073/pnas.1207230109 22689992 PMC3387103

[37] Hou Q Ye L Liu H Huang L Yang Q Turner JR. Lactobacillus accelerates ISCs regeneration to protect the integrity of intestinal mucosa through activation of STAT3 signaling pathway induced by LPLs secretion of IL-22. Cell Death Differ. (2018) 25:1657–70. 10.1038/s41418-018-0070-2 29459771 PMC6143595

[38] Erny D Hrabe de Angelis AL Jaitin D Wieghofer P Staszewski O David E. Host microbiota constantly control maturation function of microglia in the CNS. Nat Neurosci. (2015) 18:965–77. 10.1038/nn.4030 26030851 PMC5528863

[39] Hsiao EY McBride SW Hsien S Sharon G Hyde ER McCue T. Microbiota modulate behavioral and physiological abnormalities associated with neurodevelopmental disorders. Cell. (2013) 155:1451–63. 10.1016/j.cell.2013.11.024 24315484 PMC3897394

[40] Wang H Braun C Murphy EF Enck P. Bifidobacterium longum 1714 strain modulates brain activity of healthy volunteers during social stress. Am J Gastroenterol. (2019) 114:1152–62. 10.14309/ajg.0000000000000203 30998517 PMC6615936

[41] Bercik P Park AJ Sinclair D Khoshdel A Lu J Huang X. The anxiolytic effect of Bifidobacterium longum NCC3001 involves vagal pathways for gut-brain communication. Neurogastroenterol Motil. (2011) 23:1132–39. 10.1111/j.1365-2982.2011.01796.x 21988661 PMC3413724

[42] Bravo JA Forsythe P Chew MV Escaravage E Savignac HM Dinan TG. Ingestion of lactobacillus strain regulates emotional behavior and central GABA receptor expression in a mouse via the vagus nerve. Proc Natl Acad Sci USA. (2011) 108:16050–55. 10.1073/pnas.1102999108 21876150 PMC3179073

[43] Liang S Wang T Hu X Luo J Li W Wu X. Administration of lactobacillus helveticus NS8 improves behavioral, cognitive, biochemical aberrations caused by chronic restraint stress. Neuroscience. (2015) 310:561–77. 10.1016/j.neuroscience.2015.09.033 26408987

[44] Cryan JF Dinan TG Mind-altering microorganisms: the impact of the gut microbiota on brain and behaviour. Nat Rev Neurosci. (2012) 13:701–12. 10.1038/nrn3346 22968153

[45] Tillisch K Labus J Kilpatrick L Jiang Z Stains J Ebrat B. Consumption of fermented milk product with probiotic modulates brain activity. Gastroenterology. (2013) 144:1394–401. 10.1053/j.gastro.2013.02.043 23474283 PMC3839572

[46] Pirbaglou M Katz J de Souza RJ Stearns JC Motamed M Ritvo P. Probiotic supplementation can positively affect anxiety and depressive symptoms: a systematic review of randomized controlled trials. Nutr Res. (2016) 36:889–98. 10.1016/j.nutres.2016.06.009 27632908

[47] Aagaard K Ma J Antony KM Ganu R Petrosino J Versalovic J The placenta harbors a unique microbiome. Sci Transl Med. (2014) 6:237ra265 10.1126/scitranslmed.3008599 PMC4929217 24848255

[48] Jakobsson HE Abrahamsson TR Jenmalm MC Harris K Quince C Jernberg C. Decreased gut microbiota diversity, delayed bacteroidetes colonisation and reduced Th1 responses in infants delivered by caesarean section. Gut. (2014) 63:559–66. 10.1136/gutjnl-2012-303249 23926244

[49] Hooper LV. Bacterial contributions to mammalian gut development. Trends Microbiol. (2004) 12:129–34. 10.1016/j.tim.2004.01.001 15001189

[50] Gensollen T Iyer SS Kasper DL Blumberg RS. How colonization by microbiota in early life shapes the immune system. Science. (2016) 352:539–44. 10.1126/science.aad9378 27126036 PMC5050524

[51] Yan F Liu L Cao H Moore DJ Washington MK Wang B. Neonatal colonization of mice with LGG promotes intestinal development and decreases susceptibility to colitis in adulthood. Mucosal Immunol. (2016) 10:117–27. 10.1038/mi.2016.43 27095077 PMC5073052

[52] Patel RM Myers LS Kurundkar AR Maheshwari A Nusrat A Lin PW. Probiotic bacteria induce maturation of intestinal claudin 3 expression and barrier function. Am J Pathol. (2012) 180:626–35. 10.1016/j.ajpath.2011.10.025 22155109 PMC3349863

[53] Preidis GA Saulnier DM Blutt SE Mistretta TA Riehle KP Major AM. Probiotics stimulate enterocyte migration and microbial diversity in the neonatal mouse intestine. FASEB J. (2012) 26:1960–69. 10.1096/fj.10-177980 22267340 PMC3336785

[54] Schwarzer M Makki K Storelli G Machuca-Gayet I Srutkova D Hermanova P. *Lactobacillus plantarum* strain maintains growth of infant mice during chronic undernutrition. Science. (2016) 351:854–57. 10.1126/science.aad8588 26912894

[55] Preidis GA Hill C Guerrant RL Ramakrishna BS Tannock GW Versalovic J. Probiotics, enteric and diarrheal diseases, global health. Gastroenterology. (2011) 140:8–14. 10.1053/j.gastro.2010.11.010 21075108 PMC3417817

[56] Guandalini S. Probiotics for prevention and treatment of diarrhea. J Clin Gastroenterol. (2011) 45:S149–53. 10.1097/MCG.0b013e3182257e98 21992955

[57] Floch MH Walker WA Sanders ME Nieuwdorp M Kim AS Brenner DA. Recommendations for probiotic use−2015 update: proceedings and consensus opinion. J Clin Gastroenterol. (2015) 49(Suppl. 1):S69–73. 10.1097/MCG.0000000000000420 26447969

[58] Sanders ME Guarner F Guerrant R Holt PR Quigley EM Sartor RB. An update on the use and investigation of probiotics in health and disease. Gut. (2013) 62:787–96. 10.1136/gutjnl-2012-302504 23474420 PMC4351195

[59] Costeloe K Hardy P Juszczak E Wilks M Millar MR Probiotics in Preterm Infants Study Collaborative G. *Bifidobacterium breve* BBG-001 in very preterm infants: a randomised controlled phase 3 trial. Lancet. (2016) 387:649–60. 10.1016/S0140-6736(15)01027-2 26628328

[60] Jacobs SE Tobin JM Opie GF Donath S Tabrizi SN Pirotta M. Probiotic effects on late-onset sepsis in very preterm infants: a randomized controlled trial. Pediatrics. (2013) 132:1055–62. 10.1542/peds.2013-1339 24249817

[61] Kuang L Jiang Y. Effect of probiotic supplementation in pregnant women: a meta-analysis of randomised controlled trials. Br J Nutr. (2020) 123:870–80. 10.1017/S0007114519003374 31856928

[62] Xiong T Maheshwari A Neu J Ei-Saie A Pammi M. An overview of systematic reviews of randomized-controlled trials for preventing necrotizing enterocolitis in preterm infants. Neonatology. (2020) 117:46–56. 10.1159/000504371 31838477

[63] Good M Sodhi CP Ozolek JA Buck RH Goehring KC Thomas DL. *Lactobacillus rhamnosus* HN001 decreases the severity of necrotizing enterocolitis in neonatal mice and preterm piglets: evidence in mice for a role of TLR9. Am J Physiol Gastrointest Liver Physiol. (2014) 306:G1021–32. 10.1152/ajpgi.00452.2013 24742987 PMC4042115

[64] Meng D Zhu W Ganguli K Shi HN Walker WA. Anti-inflammatory effects of bifidobacterium longum subsp infantis secretions on fetal human enterocytes are mediated by TLR-4 receptors. Am J Physiol Gastrointest Liver Physiol. (2016) 311:G744–53. 10.1152/ajpgi.00090.2016 27562058 PMC5142200

[65] Meng D Sommella E Salviati E Campiglia P Ganguli K Djebali K. Indole-3-lactic acid, a metabolite of tryptophan, secreted by bifidobacterium longum subspecies infantis is anti-inflammatory in the immature intestine. Pediatr Res. (2020). 10.1038/s41390-019-0740-x. [Epub ahead of print]. 31945773 PMC7363505

[66] Xavier RJ Podolsky DK. Unravelling the pathogenesis of inflammatory bowel disease. Nature. (2007) 448:427–34. 10.1038/nature06005 17653185

[67] Ng SC Shi HY Hamidi N Underwood FE Tang W Benchimol EI. Worldwide incidence and prevalence of inflammatory bowel disease in the 21st century: a systematic review of population-based studies. Lancet. (2018) 390:2769–78. 10.1016/S0140-6736(17)32448-0 29050646

[68] Clark M Colombel JF Feagan BC Fedorak RN Hanauer SB Kamm MA. American gastroenterological association consensus development conference on the use of biologics in the treatment of inflammatory bowel disease, June 21-23, 2006. Gastroenterology. (2007) 133:312–39. 10.1053/j.gastro.2007.05.006 17631151

[69] Frank DN St Amand AL Feldman RA Boedeker EC Harpaz N Pace NR. Molecular-phylogenetic characterization of microbial community imbalances in human inflammatory bowel diseases. Proc Natl Acad Sci USA. (2007) 104:13780–5. 10.1073/pnas.0706625104 17699621 PMC1959459

[70] Haberman Y Tickle TL Dexheimer PJ Kim MO Tang D Karns R. Pediatric crohn disease patients exhibit specific ileal transcriptome and microbiome signature. J Clin Invest. (2014) 124:3617–33. 10.1172/JCI75436 25003194 PMC4109533

[71] Kaur L Gordon M Baines PA Iheozor-Ejiofor Z Sinopoulou V Akobeng AK. Probiotics for induction of remission in ulcerative colitis. Cochrane Database Syst Rev. (2020) 3:CD005573. 10.1002/14651858.CD005573.pub3 32128795 PMC7059959

[72] Miele E Pascarella F Giannetti E Quaglietta L Baldassano RN Staiano A. Effect of a probiotic preparation (VSL#3). on induction and maintenance of remission in children with ulcerative colitis. Am J Gastroenterol. (2009) 104:437–43. 10.1038/ajg.2008.118 19174792

[73] Bibiloni R Fedorak RN Tannock GW Madsen KL Gionchetti P Campieri M. VSL#3 probiotic-mixture induces remission in patients with active ulcerative colitis. Am J Gastroenterol. (2005) 100:1539–46. 10.1111/j.1572-0241.2005.41794.x 15984978

[74] Kruis W Fric P Pokrotnieks J Lukas M Fixa B Kascak M. Maintaining remission of ulcerative colitis with the probiotic *Escherichia coli* nissle 1917 is as effective as with standard mesalazine. Gut. (2004) 53:1617–23. 10.1136/gut.2003.037747 15479682 PMC1774300

[75] Tursi A Brandimarte G Papa A Giglio A Elisei W Giorgetti GM. Treatment of relapsing mild-to-moderate ulcerative colitis with the probiotic VSL#3 as adjunctive to a standard pharmaceutical treatment: a double-blind, randomized, placebo-controlled study. Am J Gastroenterol. (2010) 105:2218–27. 10.1038/ajg.2010.218 20517305 PMC3180711

[76] Gionchetti P Rizzello F Venturi A Brigidi P Matteuzzi D Bazzocchi G. Oral bacteriotherapy as maintenance treatment in patients with chronic pouchitis: a double-blind, placebo-controlled trial [see comments]. Gastroenterology. (2000) 119:305–9. 10.1053/gast.2000.9370 10930365

[77] Prantera C Scribano ML Falasco G Andreoli A Luzi C. Ineffectiveness of probiotics in preventing recurrence after curative resection for crohn's disease: a randomised controlled trial with Lactobacillus GG. Gut. (2002) 51:405–9. 10.1136/gut.51.3.405 12171964 PMC1773351

[78] Marteau P Lemann M Seksik P Laharie D Colombel JF Bouhnik Y. Ineffectiveness of lactobacillus johnsonii LA1 for prophylaxis of postoperative recurrence in crohn's disease: a randomised, double blind, placebo controlled GETAID trial. Gut. (2006) 55:842–47. 10.1136/gut.2005.076604 16377775 PMC1856210

[79] Van Gossum A Dewit O Louis E de Hertogh G Baert F Fontaine F. Multicenter randomized-controlled clinical trial of probiotics (lactobacillus johnsonii, LA1). on early endoscopic recurrence of crohn's disease after lleo-caecal resection. Inflamm Bowel Dis. (2007) 13:135–42. 10.1002/ibd.20063 17206696

[80] Lichtenstein L Avni-Biron I Ben-Bassat O. Probiotics and prebiotics in crohn's disease therapies. Best Pract Res Clin Gastroenterol. (2016) 30:81–88. 10.1016/j.bpg.2016.02.002 27048899

[81] Sears CL Garrett WS. Microbes, microbiota, colon cancer. Cell Host Microbe. (2014) 15:317–28. 10.1016/j.chom.2014.02.007 24629338 PMC4003880

[82] Abreu MT Peek RM Jr. Gastrointestinal malignancy and the microbiome. Gastroenterology. (2014) 146:1534–46.e1533. 10.1053/j.gastro.2014.01.001 24406471 PMC3995897

[83] Eslami M Yousefi B Kokhaei P Hemati M Nejad ZR Arabkari V. Importance of probiotics in the prevention and treatment of colorectal cancer. J Cell Physiol. (2019) 234:17127–43. 10.1002/jcp.28473 30912128

[84] Nowak A Paliwoda A Blasiak J. Anti-proliferative, pro-apoptotic and anti-oxidative activity of lactobacillus and bifidobacterium strains: a review of mechanisms and therapeutic perspectives. Crit Rev Food Sci Nutr. (2019) 59:3456–67. 10.1080/10408398.2018.1494539 30010390

[85] Konishi H Fujiya M Tanaka H Ueno N Moriichi K Sasajima J. Probiotic-derived ferrichrome inhibits colon cancer progression via JNK-mediated apoptosis. Nat Commun. (2016) 7:12365. 10.1038/ncomms12365 27507542 PMC4987524

[86] Talero E Bolivar S Avila-Roman J Alcaide A Fiorucci S Motilva V. Inhibition of chronic ulcerative colitis-associated adenocarcinoma development in mice by VSL#3. Inflamm Bowel Dis. (2015) 21:1027–37. 10.1097/MIB.0000000000000346 25793324

[87] Arthur JC Gharaibeh RZ Uronis JM Perez-Chanona E Sha W Tomkovich S. VSL#3 probiotic modifies mucosal microbial composition but does not reduce colitis-associated colorectal cancer. Sci Rep. (2013) 3:2868. 10.1038/srep02868 24100376 PMC3792409

[88] Ishikawa H Akedo I Otani T Suzuki T Nakamura T Takeyama I. Randomized trial of dietary fiber and *Lactobacillus casei* administration for prevention of colorectal tumors. Int J Cancer. (2005) 116:762–67. 10.1002/ijc.21115 15828052

[89] Rafter J Bennett M Caderni G Clune Y Hughes R Karlsson PC. Dietary synbiotics reduce cancer risk factors in polypectomized and colon cancer patients. Am J Clin Nutr. (2007) 85:488–96. 10.1093/ajcn/85.2.488 17284748

[90] Sivan A Corrales L Hubert N Williams JB Aquino-Michaels K Earley ZM. Commensal bifidobacterium promotes antitumor immunity and facilitates anti-PD-L1 efficacy. Science. (2015) 350:1084–89. 10.1126/science.aac4255 26541606 PMC4873287

[91] Vetizou M Pitt JM Daillere R Lepage P Waldschmitt N Flament C. Anticancer immunotherapy by CTLA-4 blockade relies on the gut microbiota. Science. (2015) 350:1079–84. 10.1126/science.aad1329 26541610 PMC4721659

[92] Li R Zhang Y Polk DB Tomasula PM Yan F Liu L. Preserving viability of lactobacillus rhamnosus GG *in vitro* and *in vivo* by a new encapsulation system. J Control Release. (2016) 230:79–87. 10.1016/j.jconrel.2016.04.009 27063422 PMC4861679

[93] Sanders ME. Impact of probiotics on colonizing microbiota of the gut. J Clin Gastroenterol. (2011) 45:S115–119. 10.1097/MCG.0b013e318227414a 21992949

[94] Maldonado-Gomez MX Martinez I Bottacini F O'Callaghan A Ventura M van Sinderen D. Stable engraftment of bifidobacterium longum AH1206 in the human gut depends on individualized features of the resident microbiome. Cell Host Microbe. (2016) 20:515–26. 10.1016/j.chom.2016.09.001 27693307

[95] Dominguez-Bello MG Costello EK Contreras M Magris M Hidalgo G Fierer N. Delivery mode shapes the acquisition and structure of the initial microbiota across multiple body habitats in newborns. Proc Natl Acad Sci USA. (2010) 107:11971–5. 10.1073/pnas.1002601107 20566857 PMC2900693

[96] Hansen CH Nielsen DS Kverka M Zakostelska Z Klimesova K Hudcovic T. Patterns of early gut colonization shape future immune responses of the host. PLoS ONE. (2012) 7:e34043. 10.1371/journal.pone.0034043 22479515 PMC3313961

[97] Suez J Zmora N Zilberman-Schapira G Mor U Dori-Bachash M Bashiardes S. Post-antibiotic gut mucosal microbiome reconstitution is impaired by probiotics improved by autologous FMT. Cell. (2018) 174:1406–23.e1416. 10.1016/j.cell.2018.08.047 30193113

[98] Strober W Fuss I Mannon P. The fundamental basis of inflammatory bowel disease. J Clin Invest. (2007) 117:514–21. 10.1172/JCI30587 17332878 PMC1804356

[99] Jostins L Ripke S Weersma RK Duerr RH McGovern DP Hui KY. Host-microbe interactions have shaped the genetic architecture of inflammatory bowel disease. Nature. (2012) 491:119–24. 10.1038/nature11582 23128233 PMC3491803

[100] Machiels K Joossens M Sabino J De Preter V Arijs I Eeckhaut V. A decrease of the butyrate-producing species Roseburia hominis and faecalibacterium prausnitzii defines dysbiosis in patients with ulcerative colitis. Gut. (2014) 63:1275–83. 10.1136/gutjnl-2013-304833 24021287

[101] Quevrain E Maubert MA Michon C Chain F Marquant R Tailhades J. Identification of an anti-inflammatory protein from faecalibacterium prausnitzii, a commensal bacterium deficient in crohn's disease. Gut. (2016) 65:415–25. 10.1136/gutjnl-2014-307649 26045134 PMC5136800

[102] Sokol H Pigneur B Watterlot L Lakhdari O Bermudez-Humaran LG Gratadoux JJ. *Faecalibacterium prausnitzii* is an anti-inflammatory commensal bacterium identified by gut microbiota analysis of crohn disease patients. Proc Natl Acad Sci USA. (2008) 105:16731–6. 10.1073/pnas.0804812105 18936492 PMC2575488

[103] Sun Z Harris HM McCann A Guo C Argimon S Zhang W. Expanding the biotechnology potential of lactobacilli through comparative genomics of 213 strains and associated genera. Nat Commun. (2015) 6:8322. 10.1038/ncomms9322 26415554 PMC4667430

[104] Donaldson GP Lee SM Mazmanian SK. Gut biogeography of the bacterial microbiota. Nat Rev Microbiol. (2016) 14:20–32. 10.1038/nrmicro3552 26499895 PMC4837114

[105] Spor A Koren O Ley R. Unravelling the effects of the environment and host genotype on the gut microbiome. Nat Rev Microbiol. (2011) 9:279–90. 10.1038/nrmicro2540 21407244

[106] Mariman R Kremer B Koning F Nagelkerken L. The probiotic mixture VSL#3 mediates both pro- and anti-inflammatory responses in bone marrow-derived dendritic cells from C57BL/6 and BALB/c mice. Br J Nutr. (2014) 112:1088–97. 10.1017/S000711451400169X 25181025

[107] Danino T Prindle A Kwong GA Skalak M Li H Allen K. Programmable probiotics for detection of cancer in urine. Sci Transl Med. (2015) 7:289ra284. 10.1126/scitranslmed.aaa3519 26019220 PMC4511399

[108] Choi HJ Ahn JH Park SH Do KH Kim J Moon Y. Enhanced wound healing by recombinant *Escherichia coli* nissle 1917 via human epidermal growth factor receptor in human intestinal epithelial cells: therapeutic implication using recombinant probiotics. Infect Immun. (2012) 80:1079–87. 10.1128/IAI.05820-11 22184415 PMC3294649

[109] Praveschotinunt P Duraj-Thatte AM Gelfat I Bahl F Chou DB Joshi NS. Engineered E. coli Nissle 1917 for the delivery of matrix-tethered therapeutic domains to the gut. Nat Commun. (2019) 10:5580. 10.1038/s41467-019-13336-6 31811125 PMC6898321

